# 

**Published:** 1882-07

**Authors:** 


### CONTENTS.

### *MISCELLANEOUS.*

PAGE.

Discussions in Homœopathic Societies and Treatment of Intermittent Fever. P. P. Wells, M. D., Brooklyn, N. Y., . . . . 289
Provings of Lachesis. B. Fincke, M. D., Brooklyn, N. Y., . . 298
Dyspnœa on Falling Asleep. E. A. Ballard, M. D., Chicago, . . 303
Clinical Reflections. Ad. Lippe, M. D., Philadelphia, . . . . 304
"The Single Remedy." P. P. Wells, M. D., Brooklyn, N. Y., . . 307
Norton's Ophthalmic Therapeutics. G. H. Clark, M. D., Germantown, 316

### *CLINICAL BUREAU:*

Cases. E. Rushmore, M. D., Plainfield, N. J., . . . . . . 318

Cures of Animals. C. F. Nichols, M. D., Boston, . . . . . . 319

### *BOOKS:*

Burnett. Supersalinity of Blood, etc. Homœopathic Publishing Company. Bœricke & Tafel, . . . . . . . . . 320

Bœricke & Tafel. The American Pharmacopœia, . . . . . 320

Appendix. Index of Cough Symptoms. . . . . . . . 3-16

